# Introducing the L.A.U.N.C.H. Collaborative

**DOI:** 10.13023/jah.0201.01

**Published:** 2020-01-26

**Authors:** F. Douglas Scutchfield, Kevin Patrick

**Affiliations:** University of Kentucky, scutch@uky.edu; University of California San Diego, kpatrick@ucsd.edu

**Keywords:** Appalachia, L.A.U.N.C.H. Collaborative, cancer, rural health, communication, broadband connectivity, health care

## Abstract

The L.A.U.N.C.H. Collaborative: Linking & Amplifying User-Centered Networks through Connected Health: A Demonstration of Broadband-Enabled Connected Health and Community-Based Co-Design brings together a group of organizations that are eager to use Appalachian Kentucky as a site for the development of a project aimed at creating an environment that addresses two of the nation’s major concerns about cancer. First, individuals who live in rural and remote areas are more likely to die of cancer than those who live in urban or suburban settings. And second, geographic obstacles hinder their ability to access evidence-based strategies that can prevent cancer or treat it once it is diagnosed.

There are those occasions that auger well for the future of various enterprises. As Shakespeare wrote:

There is a tide in the affairs of men, which taken at the flood, leads on to fortune. Omitted, all the voyage of their life is bound in shallows and in miseries. On such a full sea are we now afloat. And we must take the current when it serves or lose our ventures.

The *Journal of Appalachian Health* has found a tide that, in addition to the Journal’s fortune, also presages a potential for fortune for cancer patients in Appalachia and rural areas across America. In November 2017, we had the opportunity to participate in a session sponsored by the National Cancer Institute (NCI). This meeting focused on a new initiative that included as partners in a public–private venture the NCI, the Federal Communications Commission (FCC), Amgen, colleagues from the University of California San Diego’s Design Lab, and the University of Kentucky’s Markey Cancer Center.

This was an early planning meeting for the L.A.U.N.C.H. Collaborative: “Linking & Amplifying User-Centered Networks through Connected Health: A Demonstration of Broadband-Enabled Connected Health and Community-Based Co-Design.” (See Additional Files for full report.) This project brings together a group of organizations that are eager to use Appalachian Kentucky as a site for the development of innovative approaches to address two of the nation’s major concerns about cancer. First, individuals who live in rural and remote areas are more likely to die of cancer than those who live in urban or suburban settings. And second, geographic obstacles hinder their ability to gain access to evidence-based strategies that can prevent cancer or treat it once it is diagnosed.

Moreover, rural residents live in areas where telecommunication (broadband) capacity is either poor or non-existent. This creates concerns about their ability to engage in the prevention, diagnosis, treatment, and rehabilitation of rural cancer patients who are dependent on broadband.^1^ Since that 2017 meeting, L.A.U.N.C.H. Collaborative partners have been engaged in pilot studies that deploy new broadband-based approaches to better manage cancer. Importantly, these projects are grounded in methods of *user-centered design*^2^ that engage the residents of these communities in the co-creation of approaches to address cancer that meet their unique cultural, social, and emotional needs.

The potential value of improving broadband is a common theme in rural Kentucky beyond the aspirations of the L.A.U.N.C.H. Collaborative. An example of this is Project SOAR, Shaping Our Appalachian Region. Project SOAR was initially established by Congressman Hal Rogers of the Fifth Congressional District and Former Governor Steve Breshear and continued to prosper with support from Former Governor Bevin. A major priority of the SOAR initiative is the creation of broadband connectivity throughout Appalachian Kentucky to facilitate economic development. Many of these benefits will likely accrue to those in the healthcare sector.

The melding of these two development streams represents a supportive environment for the partners in L.A.U.N.C.H. to demonstrate the mechanisms that allow for connectivity to affect cancer prevention, treatment, and rehabilitation. This is an exciting adventure on the cutting edge of health technology. In this environment, the implementation of planned programs will be examined and judged for their effectiveness and efficiency.

The team responsible for the development of L.A.U.N.C.H. has turned to us, the *Journal of Appalachian Health*, to be a major conduit for the dissemination of the results of their early work. We will be working with them to provide a platform for both the publication of research papers and of reports that examine the nature and results of the work this consortium is doing to make a difference in the lives of cancer patients in Appalachia. We will also provide L.A.U.N.C.H. with the capacity to store, on our website, more extensive material that undergirds their work to provide guidance to others who wish to adapt and/or scale up the work done in Appalachia to other rural environments.

With our next few issues, we will bring to our readers information from the project architects, its activities, the successes and failures that it experiences, and lessons learned from the pilot projects. Importantly for this Journal, this new information will be focused specifically on cancer in Appalachia and will help outline how the use of broadband and its associated technologies can improve not only cancer care but other health problems that compromise quality of life and overall community health. Our hope is that in short order we will become an important resource for those who are engaged in rural cancer control and cancer care-based research for the roughly 25% of Americans who live in rural and remote areas.

As an editorial staff, we are delighted at this opportunity and hope that our readers share our excitement. We are anxious to make this breaking knowledge available to those who can and will use it to make a difference in cancer morbidity and mortality or recognize its applicability to other health problems of rural America. And we welcome reader comments and input into this new development and believe that it shows the importance of this Journal to the region we serve and to a broader constituency concerned with health in rural and underserved areas.
